# Validity of self-reported diseases from health surveys: comparisons with registry data in Denmark

**DOI:** 10.1093/eurpub/ckac129.426

**Published:** 2022-10-25

**Authors:** H Rosendahl, CJ Lau, M Davidsen, O Ekholm, AI Christensen

**Affiliations:** 1 Danish National Institute of Public Health, Copenhagen K, Denmark; 2 Center for Clinical Research and Prevention, Frederiksberg, Denmark

## Abstract

**Background:**

The validity of self-reported disease prevalence estimates in health surveys may be low when compared to data from medical records in administrative registers. Such discrepancies reflect a low content validity of the survey question, which may ultimately compromise the application of these survey data for public health purposes. The aim of the present study was to examine the agreement of self-reports of seven diseases with data from administrative registers, both overall and by sociodemographic characteristics.

**Methods:**

Prevalence estimates of self-reported current and/or previous diabetes, asthma, rheumatoid arthritis, osteoporosis, myocardial infarction, apoplexy, and cancer, respectively, were derived from the Danish National Health Survey in 2017 (n = 183,372 adults aged ≥16 years). Individual-level data were linked to registry data on the same diseases. Sensitivity, specificity, positive predictive value (PPV), negative predictive value (NPV), kappa, and total agreement between self-reported and registry-documented prevalence estimates were examined.

**Results:**

For all included diseases, the specificity was >92%, and the sensitivity varied between 59% (cancer) and 95% (diabetes). NPV was >94% for all diseases and PPV varied between 13% (rheumatoid arthritis) and 93% (cancer). Total agreement varied between 91 % (asthma) and 99% (diabetes), whereas kappa was lowest for rheumatoid arthritis (0.21) and highest for diabetes (0.88). Sociodemographic variables were significantly associated with total agreement with sex, age, and educational level exhibiting the strongest associations.

**Conclusions:**

Overall, total agreement, specificity, and NPV between self-reported and registry-documented disease prevalence estimates are high, but PPV and kappa vary greatly between diseases. The latter findings reflect a low content validity of the applied survey question for specific diseases. This should be taken into account when interpreting similar results from surveys.

**Key messages:**

• The validity of self-reported disease prevalence estimates may be low when compared to data from medical records. We found positive predictive values and kappa to vary greatly between diseases.

• Future studies should aim at designing survey questions properly in order to ensure a high content validity of the applied question.

